# Children's understanding of financial literacy and parents' choice of financial knowledge learning methods in Malaysia

**DOI:** 10.1016/j.mex.2023.102383

**Published:** 2023-09-16

**Authors:** Logasvathi Murugiah, Rusmawati Ismail, Hasniza Mohd Taib, Shri Dewi Applanaidu, Muhamad Noor Habibi B.Hj. Long

**Affiliations:** a Banking Department, School of Economics, Finance and Banking, College of Business, Universiti Utara Malaysia, 06010, Kedah, Malaysia; b Finance Department, School of Economics, Finance and Banking, College of Business, Universiti Utara Malaysia, 06010, Kedah, Malaysia; c Economics and Agribusiness Department, School of Economics, Finance and Banking, College of Business, Universiti Utara Malaysia, 06010, Kedah, Malaysia; d Muamalat and Halal Management Department, School of Islamic Business College of Business, Universiti Utara Malaysia, 06010, Kedah, Malaysia

**Keywords:** Financial literacy, Financial education, Primary school, Learning method, Malaysia, *Multiple Regression Analysis*

## Abstract

Financial literacy is an essential lifelong skill that should be taught to children at any age. It holds the key to develop a generation of adults who are knowledgeable about money and the economy. Additionally, OECD (2018) suggests that using digital tools could significantly enhance financial literacy and well-being. Therefore, this paper aims to:

(i) assess the financial literacy level of primary school children in the northern region of Malaysia and
(ii) explore interactive and engaging methods for teaching financial literacy.

The sample size was determined using Krejcie and Morgan's (1970) approach, resulting in 419 primary school students aged 7 to 12 and their parents. An online questionnaire was employed, and multi-regression analysis was conducted.

The findings highlighted those primary students displayed a high level of financial literacy, scoring above 80 % on the questionnaire. Furthermore, parents expressed a preference for their children to enroll in personal finance subjects offered by schools, have financial assignments or activities at school, and engage in online financial games. The study emphasized the crucial roles of schools, teachers, and active parental involvements to enhance financial literacy. This study recommends incorporating interactive and attractive teaching methods through in-class and online activities at the school level.

## Graphical abstract

**Figure:**
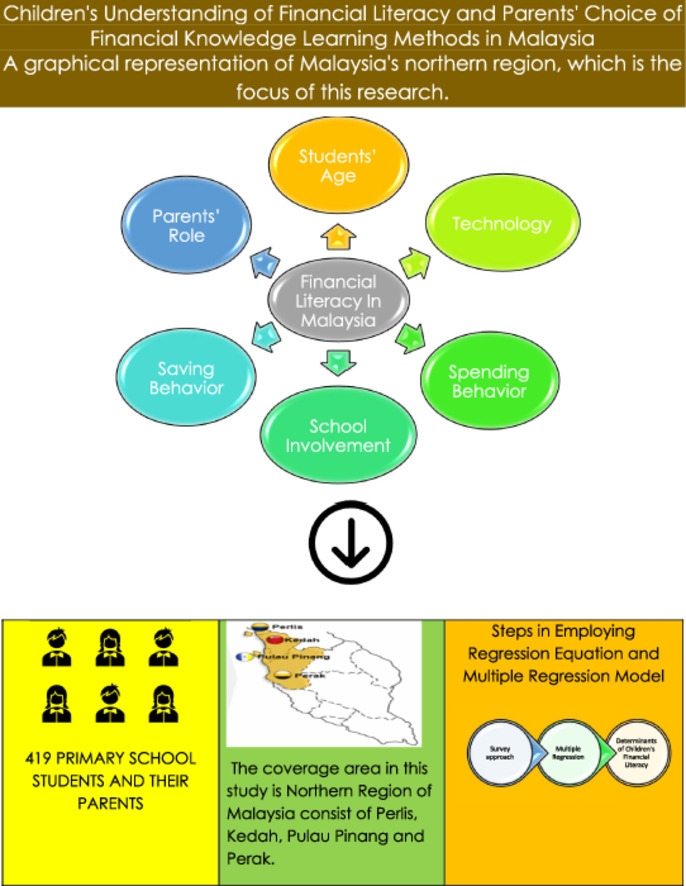

Specifications Table

**Table:** 

Subject area:	Economics and Finance
More specific subject area:	*Personal Financial Management: Financial Literacy*
Name of your method:	*Multiple Regression Analysis*
Name and reference of original method:	*J.E. Alekam, M. Md. Salleh, S. Mohd. Mokhtar, The Effect of Family, Peer, Behavior, Saving and Spending Behavior on Financial Literacy among Young Generations, AIMI 7(3) (2018) 309–323.* *OECD, OECD Principles and Good Practices for Financial Education and Awareness - Financial Stability Board. Recommendation of the Council, July 7(2005). Retrieved from*http://www.financialstabilityboard.org/2005/06/cos_050622/*.*
Resource availability:	*NA*

**Method details**

## Introduction

The issue of financial illiteracy affects Malaysians. According to research by S&P Global Literacy Financial in 2015 [1], Malaysia's financial literacy rate is only 36 %, compared to 59 % in developed nations. Furthermore, a Bank Negara Malaysia survey reveals that many Malaysians are still unable to make responsible financial decisions for their personal well-being. Many young individuals nowadays have a stronger desire to follow the most recent digital lifestyle trends, which leads to increased borrowing, personal loans, and credit card indebtedness. They would suffer with significant debt because of these habits. Additionally, the majority of young adults are susceptible to financial shocks with limited financial resilience. According to the survey, only 40 % of Malaysians believed they were financially prepared for retirement, and the rest of Malaysians do not engage in long-term financial planning. Additionally, without the ability to make prudent financial decisions, many of them are vulnerable to financial fraud and are drawn to "get rich quick" scams like the Ponzi Scheme. Undoubtedly, these shortsighted tendencies affect their development and wellbeing.

One of the best solutions to overcome this issue is to equip Malaysians with financial literacy skill. Financial literacy is the knowledge of how money works in the world. It inculcates positive values, such as saving before spending, the importance of working hard and honestly, spending wisely by distinguishing needs and wants, and giving back to the community. It is imperative for both adults and children to learn about making prudent financial decisions. The global importance of financial education for students has been established through efforts of international organizations such as the OECD [2] that had recommended that “Financial education should begin at school. People should be educated about financial matters as early as possible in their lives.” According to a Cambridge University study, children start developing money habits as early as age 7. They will eventually become aware of and pick up on their parents' spending patterns at this age. Financial education experts have long argued that introducing financial literacy to children at a young age has increased their awareness of the importance of managing their money wisely and laid the groundwork for them to develop the skills, knowledge, and habits they will need when facing financial challenges in the near future.

Undoubtedly, enhancing financial literacy is crucial to ensure that future generations of Malaysians possess the necessary knowledge and skills to effectively manage their finances. The advancement in financial literacy not only benefits individuals and their families but also has a cascading effect, propelling the entire nation towards a promising future, equipped to face the challenges that lie ahead. The overall population in Southeast Asia, particularly in Malaysia, are totally aware of the advantages of financial education and displays considerable interest in enhancing their financial literacy and money management skills. However, there is a significant disparity between the recognition of the importance of financial education and the actual implementation of effective measures to address it. Numerous financial education programs in Malaysia often adopt a short-term approach, relying on brief workshops, books, and activities that may be fun but lacking in cultivating mindset and attitude, fostering habit formation, and conducting a comprehensive long-term evaluation of their actual impact.

In Malaysia, Bank Negara Malaysia and the Securities Commissions Malaysia had launched the “5 Years National Strategy for Financial Literacy” from year 2019 to 2023 with an objective to increase Malaysians’ financial literacy in all life stages starting from school children to retirees. This plan is carried out after result showed that one in three Malaysians that have insufficient income of household usually has less knowledge in managing money thus become financially illiterate. In addition, BNM [3] found that one in three Malaysian working adults often borrow money to buy essential goods. Lastly, in term of handling their own finances, one in three Malaysians was believed to be lacked discipline and self – control. Based on the road map of five years shaping Malaysians to be financially literate that is meticulously planned by Bank Negara Malaysia, most of the plans were designated to nurture values in early age. An act of grasping the knowledge of managing expenses among Malaysians is the first concern in this road map. There are four actions lie under this priority which firstly is to implement the financial education into the curriculum for each level of education including pre - school, primary school and secondary school. Next is to carry out the financial education through the co - curricular activities. Establishing the capacity development and giving support for teachers is the third action which also becomes the first concern in this road map. Lastly, endless support to the students, parental groups and community will be given. Therefore, it is certainly crucial to delve into the level of financial literacy among children nowadays that one may get greater picture of how to work in raising the literacy rate among them. Hence, the issue of financial literacy was indeed a national issue.

We are convinced that the key to wiping out excess personal debt among Malaysian youth lies in early childhood education. Yet, talking about money and savings doesn't sound like fun for anyone involved, but learning about money can be fun with interactive game such as kids practice identifying, counting and saving money. The right tool would be able to build a fundamental understanding of saving while providing them with real world skills practice, develops and brings to market innovative products to help parents and educators teach kids the skills of basic personal finance. It would be great if we can see that every child in Malaysia is taught everything that he or she needs to know about money before leaving high school. Hence, new approach or educational game helps young kids learn faster. By giving our children basic financial education, they would empower and take control over their financial freedom, and their futures. This will include what money really is, how to make it, how to save it, how to account for it and how to plan for its use.

Aligned with this objective, Bank Negara Malaysia (BNM) is actively driving nationwide financial education initiatives through various means. These include the formulation of policies and the establishment of strategic directions, such as the Financial Sector Master Plan 2001–2010, the Financial Sector Blueprint 2011–2020, and the ongoing development of the Financial Education Network. Moreover, BNM integrates financial education into the school curriculum, implements school adoption programs, fosters school financial clubs, and provides adult education through the AKPK (Credit Counselling and Debt Management Agency).

BNM has also identified specific target groups and priority areas, namely school children, youths, lower-income households, and teachers, to enhance financial education in Malaysia. Recognizing the importance of financial literacy as a life skill, BNM advocates for the promotion of financial education programs throughout different stages of life, beginning from early childhood. To support this, AKPK offers adult financial education programs. Additionally, BNM emphasizes the need for an effective financial education assessment framework that offers comprehensive, reliable, and timely information to support the implementation of these initiatives.

Therefore, the primary objectives of this research are as follows: (i) to assess the financial literacy levels among a group of children aged 7 to 12 attending primary school in the northern region of Malaysia, and (ii) to investigate attractive and interactive approaches for teaching financial literacy.

## Past studies

The significance of financial literacy and its fundamental components for the next generation lies in the fact that young children are the future pillars of nations and will assume leadership roles in various fields. Consequently, financial literacy plays a pivotal role in shaping their financial goals, encompassing everything from managing their pocket money to handling personal accounts [4].

Existing literature strongly emphasizes that individuals who possess higher levels of financial literacy tend to exhibit enhanced performance when engaging with numeracy-related matters [[5], [6], [7]], savings and cost-cutting [8,9], calculating earnings on the saved items [6], weighing the bank accounts with higher interest rates and profits [4], and diversifying the risk with better alternatives and risk-mitigating behavior [7,10]. Given these financial benefits, the current study argues that financial literacy plays a crucial role in the lives of young individuals by empowering them to create effective financial plans, set future financial objectives, make informed investment decisions, mitigate risks, and assess their portfolios [11,12]. Prior research emphasizes that the level of financial literacy among young individuals is influenced by various demographic factors [5,9,11,[13], [14], [15]].

Previous study by [16] claim that money attitude is a crucial factor that significantly influences the saving and spending behavior of students, thereby affecting their overall financial literacy. In their study, [17] revealed that students with high levels of confidence are more likely to effectively manage their budgets by employing appropriate budgeting techniques and strategies. This finding underscores the importance of confidence in managing financial budgets, saving money, and planning expenditures based on budgeting skills. Alternatively, students who possess lower self-control demonstrated their inclination toward emotional beliefs while designing their budgeting techniques.

Moreover, [18] highlighted the significant impact parents have on the financial well-being, knowledge, attitudes, and capabilities of emerging adults. Additionally, [19] highlighted various financial aspects within families, such as personal attributes, family characteristics, interactions among family members, and maintaining connections with relatives, which contribute to the financial literacy of students and young adults.

Furthermore, studies have revealed that social inclusion, the external environment, and peers play substantial roles in shaping the financial literacy of young children. In line with this, [20] concluded from their study that peers are considered a crucial factor when it comes to designing capital budgeting techniques and determining saving patterns.

In conclusion, [21,22] acknowledge that technological advancements and the rise of modern platforms like social media (such as Facebook, Twitter, Instagram), digital transformation, and the digital orientation of the contemporary world have a substantial impact on the financial literacy of students. These technological innovations play a significant role in shaping students' financial literacy.

Previous literature has also demonstrated the influence of financial behavior and financial socialization agents on children's financial literacy. Based on these findings, the following hypotheses have been formulated for this study.

**Table:** 

H1	Saving behavior has significant effect on children financial literacy.
H2	Spending behavior has significant effect on children financial literacy.
H3	Parent role has significant effect on children financial literacy.
H4	Peer role has significant effect on children financial literacy.
H5	School involvement has significant effect on children financial literacy.
H6	Technology has significant effect on children financial literacy.
H7	Children's gender has significant effect on their financial literacy.
H8	States in Northern region of Malaysia have significant effect on children financial literacy.
H9	Household income has significant effect on children financial literacy.
H10	Parents’ education level has significant effect on children financial literacy.
H11	Parents’ occupation has significant effect on children financial literacy.
H12	Parents’ marital status has significant effect on children financial literacy.
H13	Children's age has significant effect on their financial literacy.
H14	Race has significant effect on children financial literacy.
H15	Type of school has significant effect on children financial literacy.

## Methodology

The research framework for this study was developed according to the past literature and theory of family financial socialization and consumer socialization. According to [23] theoretical framework helps researcher to explore the validity of the theory through various analysis test towards the independent and dependent variables.

**Fig. 1:**
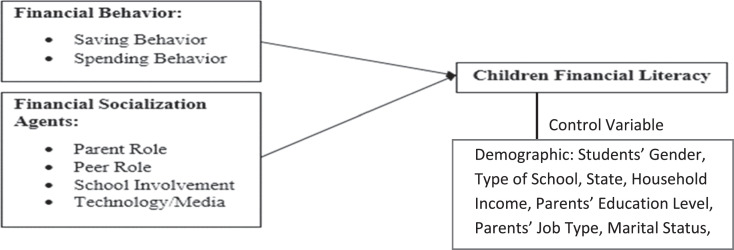
Theoretical framework.

Fig. 1 showed the proposed of theoretical or research framework for this study. In this research the theoretical framework was modified to fit with the research objectives. Based on the proposed theoretical framework as shown in Fig. 1, the independent variables of this study are financial behavior and financial socialization agents. For financial behavior, the measurement used to measure the influence towards financial literacy of the children are saving and spending behavior of the children whilst financial socialization agents are parent role, peer role, school involvement and technology/media.

**Fig. 2:**
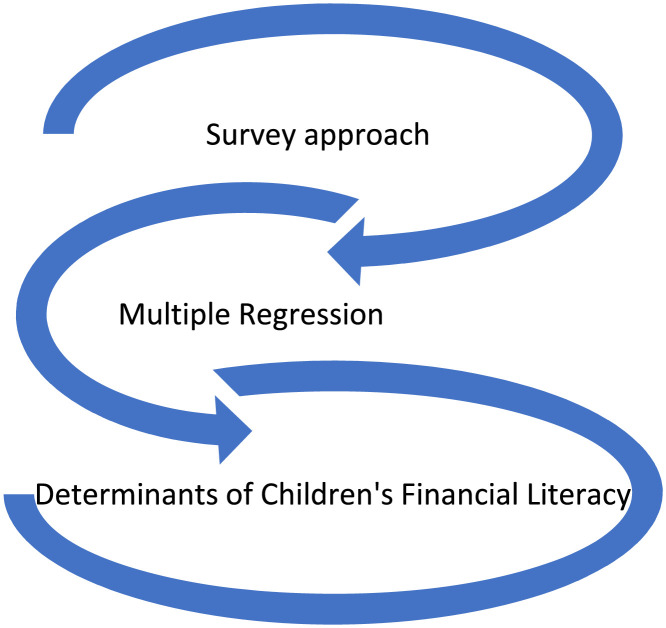
Steps in employing multiple regression model.

Quantitative methods were employed to conduct this study. The population for this study consisted of students aged 7 to 12 years old in 547 primary schools (both public and private) located in the Northern Region of Malaysia, as listed by the Ministry of Education in 2020. The total number of students in the population was 545,196, along with their parents. Fig. 2 below provides details of the selected sample.

To determine the sample size for this study, the researcher used the measurement criteria established by [24]. With a population size (N) of 545,196 students, a chi-square value (x^2^) of 3.841 was obtained at a 5 % significance level and degree of freedom equal to 1. At a 95 % confidence level, the margin of error (d^2^) was set at 0.05. The population proportion (P) was considered to be 50 %, indicating an expected chance of selection at 50 %. Based on the information derived from the formula, the estimated sample size was determined to be 384 respondents.

In order to get 384 number of respondents, simple random sampling procedures applied to cover Perlis, Kedah, Penang and Perak as shown in Table 1. The main reason of this study applied simple random sampling procedures was because population had a chance to be selected during the collection data. Besides that, simple random sampling procedures was more straightforward procedures and it reduced bias. The respondents were segregated based on state and the diversification of the respondent were derived from the proportion of the population between the states.

**Table 1:** Tabulation of primary pupils and number of students in northern region for year 2020 and sample size.

State	Number of pupils	Percentage number of students	Projected number of respondents (sample size=384 pupils)	Actual number of respondents and percentage
Perlis	24,156	4.43 %	17	35 (8.35 %)
Kedah	189,160	34.70 %	133	136 (32.46 %)
Penang	130,386	23.92 %	92	106 (25.30 %)
Perak	201,494	36.96 %	142	142 (33.89 %)
**Total**	**545,196**	**100.00 %**	**384**	**419 (100 %)**

Source: Ministry of Education Malaysia, 2020.

The actual number of respondents selected for the study was 419, which exceeded the projected sample size of 384. Specifically, for Perlis, the projected proportion was 4.43 %, equivalent to 17 respondents, but the actual number of respondents obtained was 35, representing 8.35 %. In the case of Kedah, the projected proportion was 34.70 % (133 pupils), and a total of 136 usable responses were collected, accounting for 32.46 %. The targeted sample size for Penang was 92 respondents, representing 23.92 %, but the actual number of respondents obtained was 106. Lastly, for Perak, the researcher managed to collect 142 respondents to meet the projected target. The respondents in this study were 419 children between the ages of 7 to 12 years old and their parents who resided in the states of Perlis, Kedah, Penang, and Perak for the year of 2021.

The Covid-19 pandemic, however, has caused some challenges for researchers. To comply with the movement-control order (MCO) issued by the authorities, it is necessary to adjust the data gathering method from interviews to the distribution of online surveys. Attempts were made to ensure that the responses obtained using this method are equally reliable and that the data produced are still packed with information. Additionally, using online surveys has the advantages of being quicker, less expensive, more accurate, quick to analyze, and requiring more truthful responses.

Data collection employed a survey approach by distributing a set of online questionnaires to the respondents. The questionnaire consists of 46 multiple choice questions that must be answered by the students and 9 multiple choice questions that must be answered by their parents. The level of the children's financial literacy is measured by using 15 questions. The questionnaire starts with 9 questions on the behavior and background of the parents. Subsequently, there were 10 questions about spending behavior, 7 questions about saving behavior, 4 questions about peer roles, 3 questions about school involvement, and 3 questions about technology, all these questions developed to measure independent variables as tabulated in Table 2.

**Table 2:** Questionnaire structure.

Questionnaire structure	Number of questions
Part A: parental section	**9 Questions**
• Parental characteristics	4 Questions
• Parental roles	5 Questions
Part B: students section	**46 Questions**
• Student characteristics	4 Questions
• Financial literacy/saving behavior/ spending behavior/peer roles/ school involvement/ technology	42 Questions

The questionnaires were developed through adaption of previous studies, Incharge Debt Solution (a non-profit credit counseling organization) and the organisation for Economic Co-operation and Development (OECD).

The operational definition for each variable was defined based on the understanding of the researcher based on the previous literature. Table 3 below shows the dependent and independent variables used for this study:

**Table 3:** Variables and operational definition.

Variables	Number of question	Operational definition	Reference
Dependent variables			
Financial literacy (FL)	15 Questions	Children financial literacy is a capability of young children about sharing, saving and spending that will support a good financial habits and practices.	[2] Incharge Debt Solution [34] [35] [36]
Independent variables			
Financial behavior (FB)			
Saving behavior (SAVING)	10 Questions	Saving behaviors are described as attitude of the children to save part of an income after minus their expenses.	[2] [34] [35] [36] [37] Incharge Debt Solution [38]
Spending behavior (SPENDING)	7 Questions	Spending behavior are described as an attitude of the children to spend their income to utilize their needs and wants.	[2] Incharge Debt Solution [35] [36]
Financial socialization agents (FSA)			
Parents role (PARENT)	8 Questions	Parents role are described as an attitude and characteristics of the parents that could influence their children financial literacy.	[35] [36] [39]
Peer role (PEER)	4 Questions	Peer role are described as an attitude and characteristics of the peer that could influence the children financial literacy.	[2] [25]
School Involvement (SCHOOL)	3 Questions	School Involvement are described as a participation of the school towards the children financial literacy.	[35] [39]
Technology (TECH)	3 Questions	Technology is described as a medium that usually used by the children could influence the children financial literacy.	[39]

The reliability test was performed on variables financial literacy, financial behavior and financial socialization agents yielded a Cronbach's Alpha value of 0.765. Therefore, the data had passed the reliability assumption and indicated an internal consistency.

Research design encompassed descriptive analysis, correlation analysis and multiple regression analysis. Descriptive analysis assisted researchers to identify the background of respondents and the scores for every section in the questionnaires. Meanwhile, correlation and regression analysis were performed to measure the relationship among independent variables and the dependent variable in the study. The adequacy of the model is interpreted using adjusted R^2^ and the overall F-statistics. The coefficient sign confirmed the relationship between independent variables and the dependent variable, where the existence of significant relationship between independent and dependents variables is determined by the probability values.

To generate the best output in regression analysis, researcher come up with the following steps in employing regression equation and multiple regression model used for this study. The regression equation model employs the “Stepwise Method” and “Enter Method” to identify the determinants of children's financial literacy in northern region of Malaysia as depicted in Fig. 2.

Thus, the multiple regression model takes on the following form (Table 4) as an outcome of the above process:

**Table 4:** Multiple regression model.

${FL}_{it}=\alpha +\beta_{1}SAVING\;+\;\beta_{2}SPENDING+\beta_{3}PARENT+\beta_{4}PEER+\beta_{5}SCHOOL+\beta_{6}TECH+\beta_{7}GENDER\;+\;\beta_{8}SCHOOLTYPE+\;\beta_{9}STATE+\;\beta_{10}HOUSEHOLD\;INCOME+\;\beta_{11}PEDU+\;\beta_{12}PJOB\;+\;\beta_{13}MARITAL+\;\beta_{14}SA+\;\beta_{15}RACE+\varepsilon$	
Abbreviation	Terminology
FL_it_	Financial literacy
SAVING	Saving behavior
SPENDING	Spending behavior
PARENT	Parents role
PEER	Peer role
SCHOOL	School involvement
TECH	Technology
GENDER	Students’ gender
SCHOOL TYPE	Type of school
STATE	State
HOUSEHOLD INCOME	Household income
PEDU	Parents’ education level
PJOB	Parents’ job type
MARITAL	Marital status
SA	Students’ age
RACE	Race
ε	Error term

## Findings & discussions

### Descriptive

Table 5 depicts the mean value of financial literacy level among primary students in northern region of Malaysia is at 10.10 or 84.17 % (minimum value is 4 and maximum value is 12 or 10.10/12 *100 = 84.17 %) where students can answer most of the financial literacy questions correctly. This result indicates that the primary students in northern region of Malaysia possess a high level of financial literacy since [25], [26], [27] deemed that score of more than 80 % were considered as high level of financial literacy. Meanwhile, mean values for independent variables such as (1) school involvement; (2) spending behavior, (3) saving behavior, (4) technology and (5) parents’ role show above average values. On the other extreme, peer role scores below average mean value at 2.97. Descriptive results for demographic factors such as gender, type of school, states, household income, parents’ education level, parents’ job type, marital status, students’ age, and races are also shown in Table 5.

**Table 5:** Descriptive analysis.

***N*** **= 419**	**Minimum**	**Maximum**	**Mean**	**Std. deviation**
	Statistic	Statistic	Statistic	Statistic (Std. Error = 0.238)
**Financial Literacy**	4	12	10.10	1.253 (0.238)
**Peer Role**	0	5	2.97	1.119
**School Involvement**	0	4	3.57	.924
**Spending Behaviour**	0	10	6.26	1.840
**Saving Behaviour**	0	12	9.11	1.479
**Technology**	0	3	1.73	1.041
**Parents’ Role**	0	5	3.96	.949
**Gender**	1 [male]	2 [female]	186 [male], 233 [female]	
**Type of School**	1 [urban]	2 [rural]	304 [urban], 115 [rural]	
**State**	1 [Kedah], 2 [Penang], 3 [Perak], 4 [Perlis]		134 [Kedah], 107 [Penang], 141 [Perak], 35 [Perlis]	
**Household Income**	1	10	4.94	2.591
**Parents’ Education Level**	1 [Not schooling], 2 [Primary level], 3 [PMR], 4 [SPM], 5 [Diploma/STPM/Matriculation and equivalent], 6 [Degree level], 7 [Master/PhD/Professional Certificate]		3 [Primary level], 3 [PMR], 98 [SPM], 92 [Diploma/STPM/Matriculation], 167 [Degree], 56 [Master/ PhD/ PC]	
**Parents’ Job Type**	1 [Government], 2 [Private], 3 [Self-employed]		211 [Government], 122 [Private], 86 [Self-employed]	
**Marital Status**	1 [single]	2 [married]	21 [single], 398 [married]	
**Students’ Age**	7 years old	12 years old	9.91	1.686
**Race**	1 [Malay], 2 [Chinese], 3 [Indian], 4 [Others]		295 [Malay], 43 [Chinese], 74 [Indian], 7 [Others]	

### Correlation

Table 6 suggests that financial literacy is significantly and positively correlated with all independent variables namely, spending behavior, technology, school involvement, peer role, parents’ role and saving behavior at 1 % confidence level based on Pearson Correlation analysis.

**Table 6:** Correlation analysis.

**Correlations**								
		Financial literacy	Parents’ role	Peer role	School involvement	Spending behavior	Saving behavior	Technology
Financial Literacy	*Pearson Corr.*	1						
	Sig. (2-tailed)	–						
Parents’ Role	*Pearson Corr.*	.297⁎⁎	1					
	Sig. (2-tailed)	.000	–					
Peer Role	*Pearson Corr.*	.313⁎⁎	.183⁎⁎	1				
	Sig. (2-tailed)	.000	.000					
School Involvement	Pearson Corr.	.495⁎⁎	.054	.319⁎⁎	1			
	Sig. (2-tailed)	.000	.274	.000	–			
Spending Behavior	*Pearson Corr.*	.574⁎⁎	.373⁎⁎	.403⁎⁎	.382⁎⁎	1		
	Sig. (2-tailed)	.000	.000	.000	.000	–		
Saving Behavior	*Pearson Corr.*	.290⁎⁎	.220⁎⁎	.231⁎⁎	.100*	.392⁎⁎	1	
	Sig. (2-tailed)	.000	.000	.000	.042	.000	–	
Technology	*Pearson Corr.*	.528	.192⁎⁎	.274⁎⁎	.441⁎⁎	.470⁎⁎	.135⁎⁎	1
	Sig. (2-tailed)	.000	.000	.000	.000	.000	.006	–

⁎⁎ Correlation is significant at 1 % (2-tailed). *Pearson Corr*. = Pearson correlation.

⁎ Correlation is significant at 5 % (2-tailed).

### Multiple regression analysis (Coefficient analysis)

Findings in Table 7 depict that 51.7 % of the variables can be explained as noted in R-Square result. Correspondingly, the findings interpret that Adjusted R Square (adj. R^2^) can be explained at 50.8 % to accurately report the data. The results also show that the independent variables are statistically significantly to predict the dependent variable, *F* = 54.891, p < 0.0001 (i.e., the regression model is a good fit of the data).

**Table 7:** Multiple regression analysis - coefficient analysis.

Variable	Beta (t-value)
Constant	5.701 (14.660)
Students’ Age	**0.233**⁎⁎⁎**(5.231)**
School Involvement	**0.222**⁎⁎⁎**(5.483)**
Spending Behavior	**0.236**⁎⁎⁎**(5.094)**
Technology	**0.146**⁎⁎⁎**(3.218)**
Saving Behavior	**0.103**⁎⁎**(2.667)**
Parents’ Role	**0.102**⁎⁎**(2.699)**
State	−0.045 (−1.248)
Type of School	0.036 (0.987)
Parents’ Income	0.030 (0.581)
Parents’ Job Type	0.023 (0.567)
Parents’ Marital Status	−0.018 (−0.500)
Parents’ Education	−0.009 (−0.181)
Race	0.008 (0.200)
Peer Role	0.004 (0.114)
Students’ Gender	−0.004 (−0.121)
**R-Square**	**0.517**
**Adj R-square**	**0.508**
**F-Value**	**54.891**

**DV: Financial Literacy**.

Note: * significant at 10 %.

⁎⁎⁎ significant at 1 %.

⁎⁎ significant at 5 %.

Further discussions of the findings are explained below.

Students’ Age – Students’ financial literacy shows a significant and positive relationship relevant to their age. This is evident from the sig. value of 0.000, which is lower than the 0.01 acceptable threshold. Each 1 % increase in the students' age results in a corresponding 0.233 or 23.3 % increase in their financial literacy level. These findings highlight a statistically significant difference in financial literacy among students of different age groups in the northern region of Malaysia. This study aligns with the previous research conducted by [25]. This means that there is a difference in children's financial literacy for students aged 10 to 12 years and students aged 7 to 9 years. Older age groups of children have better financial literacy than their younger counterparts. As children age, financial literacy will also increase. This is likely because older children have knowledge and experience in handling money. Apart from that, this experience can also be learned from their parents. Consequently, it is crucial to consider the curriculum and tailor it to the specific age groups. To address this, the topic can be divided into three main modules based on the learners' age and proficiency level.

School Involvement – Students’ financial literacy shows a significant and positive relationship relevant to schools’ involvement, as evidenced by the sig. value of 0.000, which is lower than the 0.01 acceptable threshold. A 1 % increase in school involvement corresponds to a 0.222 or 22.2 % increase in financial literacy levels. School involvement refers to the active participation of schools in promoting children's financial literacy. In promoting knowledge and financial literacy among kids, schools and teachers must actively participate in the process. It is strongly advised that schools and instructors actively fulfill their responsibilities and actively engage with the students to enhance the financial literacy levels of primary school pupils in the northern region of Malaysia. Furthermore, this study highlights the importance of utilizing interactive and engaging approaches in teaching financial literacy, which can be revisited through a combination of in-class activities and online resources at the school level. It emphasizes the crucial role of schools in fostering children's financial literacy. Moreover, schools have the potential to make the most significant impact on children's financial literacy, serving as the primary influencer in shaping their financial behavior and overall level of financial literacy. These findings align with previous research [28,29].

Spending Behavior – Students’ financial literacy shows a significant and positive relationship relevant to spending behavior, as indicated by the Sig. value of 0.000, which falls below the 0.01 acceptable threshold. A 1 % increase in spending behavior corresponds to a 0.236 or 23.6 % increase in financial literacy levels. Spending behavior refers to children's attitudes towards utilizing their income for their needs and wants. These findings emphasize that children's attitudes towards spending improve and become more manageable with a solid foundation in financial literacy. The prudent spending mindset instills in these students the importance of understanding and managing their expenses. Moreover, the study also highlights the inclusion of donations as an expenditure category. Respondents who demonstrate effective budget management exhibit a more favorable attitude towards financial literacy. These results are consistent with the past findings [23,30], indicating a positive relationship between financial behavior (spending and saving) and children's financial literacy.

Technology: The impact of technology on students’ financial literacy is significantly positive, supported by a sig. value of 0.000, which is below the 0.01 acceptable threshold. A 1 % increase in technology usage corresponds to a 0.146 or 14.6 % increase in financial literacy levels. Technology serves as a medium commonly used by children, influencing their financial literacy. It offers numerous avenues for improving financial literacy, such as providing access to a wide range of financial information and making financial education fun, easy to understand, and interactive. Technology plays a crucial role in the learning process, including the development of financial literacy. Greater digital literacy fosters effective financial management by facilitating easy access to financial information. These findings align with previous studies [31,32].

Saving Behavior:

The study highlights a significant positive relationship between saving behavior and financial literacy, with a sig. value of 0.05. A 1 % increase in saving behavior leads to a 0.103 or 10.3 % increase in financial literacy levels. Saving behavior refers to children's inclination to set aside a portion of their income after deducting expenses. The results demonstrate that a good understanding of financial literacy positively influences children's saving habits. Therefore, schools, policymakers, and financial institutions should prioritize enhancing financial literacy among primary students as a valuable strategy to encourage savings behavior and the intention to save. These findings align with previous studies [23,30], indicating a positive relationship between financial behavior (saving) and children's financial literacy.

Parents' Role:

The study reveals a significant positive impact of parents' role on financial literacy, with a sig. value of 0.05. A 1 % increase in parents' involvement corresponds to a 0.102 or 10.2 % increase in financial literacy levels. Parents' role encompasses their attitudes and characteristics that influence their children's financial literacy. Children acquire financial education through observation and imitation of their parents. Therefore, positive parental role models play a vital role in motivating their children's development. Parents who actively engage in raising their children tend to have a greater influence on their children's financial literacy levels. This aligns with the findings of [33], who provide strong evidence that employed parents, due to limited interaction time, can affect their children's learning processes.

For other variables, it could be concluded that there was insignificant relationship with financial literacy among primary school students at the northern region of Malaysia.

Summary of the findings for the hypotheses:

**Table:** 

H1	Accepted	H9	Rejected
H2	Accepted	H10	Rejected
H3	Accepted	H11	Rejected
H4	Rejected	H12	Rejected
H5	Accepted	H13	Accepted
H6	Accepted	H14	Rejected
H7	Rejected	H15	Rejected
H8	Rejected		

Meanwhile, for the second objective, this study finds that parents prefer their children to be enrolled in personal finance subjects offered by school (259), to be assigned with financial topics tasks or activities at school level (149) and to be involved in online financial games (143) as shown in the following Table 8.

**Table 8:** Financial learning method prefer by parents to their children.

## Conclusions

The financial literacy level of primary students in the northern region of Malaysia stands at 10.10 or 84.17 % (calculated by taking the minimum value of 4 and the maximum value of 12, resulting in 10.10/12 *100 = 84.17 %). This finding indicates that the primary students in the northern region of Malaysia exhibit a high level of financial literacy, as they can answer most of the financial literacy questions correctly. [25], [26], [27] indicates that students who score more than 80 % on the questions are considered to have a high level of financial literacy. Among the demographic factors examined, only the age of the students shows a significant and positive correlation with financial literacy. As for the independent variables, most of them, namely spending behavior, technology, school involvement, parents' role, and saving behavior, demonstrate a positive and significant relationship with financial literacy. However, peer influence does not show a significant relationship.

To begin with, teachers responsible for teaching foundational finance subjects should ensure they are well-prepared and possess a thorough understanding of the syllabus. The module should be supplemented with a range of co-curricular activities and practical learning methods, such as board games, online games, and project-based games. Additionally, it would be beneficial to raise awareness about financial education among underprivileged students in Malaysia. This could be achieved by establishing dedicated financial literacy corners in school libraries and universities, implementing financial mentor-mentee programs, and organizing industry talks on financial topics. These initiatives aim to provide accessible resources and guidance to foster financial literacy among students.

This study holds significant value as it contributes to a broader understanding and findings within the specific context. Previous research on financial literacy among children has been limited, prompting us to undertake this study with the aim to enhance children's financial literacy, specifically among primary school children in Perlis, Kedah, Penang, and Perak.

The findings of this study can be a guideline for the government, industry players, and academicians in achieving the objective set by Bank Negara Malaysia for 2023, which is to raise the level of financial literacy among Malaysians. Consequently, the results can help shape consumer behavior from the primary stages and create awareness among all relevant parties regarding the importance of increasing financial literacy among children.

Our results highlight that school involvement plays a significant role in children's financial literacy, as socialization agents have the greatest influence, followed by technology. Teachers and/or relevant parties could play a role in developing comprehensive basic financial education modules for different target groups, namely (1) pre-school, (2) primary school, (3) high school, and (4) institutions of higher learning. The modules and development support are also accompanied by teaching notes so that teachers who teach basic finance subjects are better prepared and better understand the syllabus that needs to be taught. The module is accompanied by various co-curricular activities, practical learning methods through board games, online games as well as project -based games. Finance education carnival and awareness campaign to the target group can be done by creating a financial literacy corner in school libraries and in universities, financial mentor program by trained students where students can offer basic financial knowledge guidance to other students in school, internal mentoring sessions are conducted regularly and personally as well as through appropriate social media in school, talks from trained students to target groups on financial education through positive financial behavior and knowledge, and students organize talks with the industry (e.g., AKPK, BNM, banking or other financial institutions) on financial education through positive financial behavior.

Additionally, the findings emphasize the importance of raising awareness among parents to actively engage in their children's financial education. Research has shown that employed parents may have limited interaction time, which can impact their children's learning processes [33]. Thus, it becomes evident that parents can significantly influence their children's financial literacy. Furthermore, the Ministry of Communications and Multimedia can leverage this study to collaborate with industry players such as Perbadanan Tabung Pendidikan Tinggi Nasional (PTPTN) to develop and enhance online platforms for children's financial literacy programs.

In summary, the study's outcomes in Malaysia can serve as a valuable reference point for other regions within the country and for countries facing similar challenges in financial literacy education. The recommendations and strategies can be adapted and customized to suit the unique needs and contexts of different regions and countries striving to enhance financial literacy among their youth. Hence, this study aims to assist the government, industry players, and academicians in the development of formal education curricula and co-curricular activities for primary schools, with a focus on enhancing children's financial mastery in Malaysia. It also serves to evaluate the effectiveness of the "BNM 5 Years National Strategy for Financial Literacy 2019–2023″ program, specifically among young children [3].

## Limitations and future research direction

One of the limitations of this study is the use of simple random sampling, which resulted in an unequal tabulation of respondents by race. Another limitation of the study is the geographical restriction to only northern part of Peninsular Malaysia, which limits the generalizability of the findings to a larger population. Additionally, the Covid-19 pandemic, however, has caused some challenges for researchers. To comply with the movement-control order (MCO) issued by the authorities, it is necessary to adjust the data gathering method from interviews to the distribution of online surveys.

Further research should consider using stratified sampling to ensure a more equal representation of different races in the sample. Furthermore, future studies should aim to expand the geographic scope of the research the whole states of Malaysia in order to obtain more comprehensive and meaningful results. Further, a deeper analysis of the specific areas in which students demonstrated proficiency and the areas that may require further attention would provide richer insights. This could guide educators and policymakers in tailoring interventions effectively. By addressing these limitations, future research can provide a more comprehensive understanding on the impact of financial literacy on children from different races and regions.

## Declaration of Competing Interest

The authors declare that they have no known competing financial interests or personal relationships that could have appeared to influence the work reported in this paper.

## Data Availability

The data that has been used is confidential.
